# Prognosis in Insanity

**Published:** 1898-07-30

**Authors:** F. St. J. Bullen

**Affiliations:** late Medical Officer West Riding Asylum, Wakefield


### Medical Progress and Hospital Clinics.

[*The Editor will be glad to receive offers of co-operation and contributions from members of the profession. All letters should be addressed to* The Editor, at the Office, 28 & 29, Southampton Street, Strand, London, W.C.]

### PROGNOSIS IN INSANITY.

By F. St. J. Bullen, M.R.C.S., late Medical Officer West Riding Asylum, Wakefield.

(*Continued from page* 285.)

**nfluence of Delusion.**—No symptom can lend more weight to prognosis than the above. The following points should be kept in mind in estimating the relative importance of this factor in the mental state. 1. Number: Except in the more advanced stages of enfeeblement or in acute cases, plural delusions do not usually exist. By this is not meant the many developments of a ruling idea, such as that of greatness, but varying and various insane conceptions without noticeable connection. Many delusive ideas, immature, of delirious type, are bred during acute mental disorders, and so long as they remain plural and disconnected have no serious significance. But a single prominent false concept is from the first a symptom of serious import. 2. Fixity: As has just been stated transient delusions are frequent in acute cases, and have little or no serious weight so long as they remain changeable. But any idea which persistently thrusts itself forward and above its surrounding satellites has grave significance. So that in the course, or during the subsidence, of acute disorders the uncovering of some focal delusion is a bad sign. 3. Origin: Delusions based on hallucinatory phenomena and on removable physical causes are less unfavourable than those which are founded on organic changes of a nature neither perceptible to the individual nor removable; that is, delusions indicating a break-down originating within the patient through his faulty organisation. Where, however, the delusion born of an hallucination outlasts for some time the disappearance of the latter there must be fear of its permanence. 4. The manner of evolution and rate of growth: These are most important. As with insanity generally, so with delusion in particular; a slow insidious growth has a very hopeless outlook, for it implies a destruction of old, nearly normal associations, and their replacement by a new perverted organisation. Particularly bad is the full growth of a delusion without notable emotional disturbance; the evolution of a false conception through a disturbed period of ever-weakening doubts, ending in its entire acceptance by and then persistent domination of the mind. 5. Character of delusion: Various as may be the forms which delusory ideas take, there are certain of these which are especially of threatening or hopeless prognosis, inasmuch as they signify grave alterations in normal mental constitution. Foremost amongst these is the idea of exaltation. Merely temporary and general feelings of well-being, or even grandeur, which are in harmony with the prevailing emotional tone of an acute attack are not here alluded to; but the acceptance of a distinct idea of personal exaltation which remains persistent means irrecoverable insanity. Whether it be found in the general paralytic, the epileptic, the decadence after climacteric and of senility, or in the gradual partial mental enfeeblement of the monomaniac, the outlook is the same. In the destructive process, the outcome of a welling up of self-feeling during dissolution of (intellectual) association systems, or again in the constructive form where a new nexus of ideas (woven to a pattern designed by watchful self-introspection) becomes organised, and the new self arises supreme amidst the fragments of discarded doubts and a hostile environment; in both alike, exaltation signifies mental ruin. The last form expresses, together with exaltation, a change in the condition of the personality, and this alteration in self-feeling is met with in depressed as in exalted states. Thus arise doubts concerning and even loss of, or duplex, personality, ideas of belittlement or the worst forms of hypochondriacal delusion; all such springing from perverted organic sensation. The maintenance or improvement of physical health under such conditions only renders the prognosis yet more hopeless. Where the delusion is such as to favour self-destructive tendencies, suicidal attempts, refusal of food, and general mal-nutrition must receive consideration. In this place we may note the unfavourable character of delusions of debased sexuality, with the hallucinations of common sensation and smell. Ideas of self-depreciation with an emotional tone in harmony, and apart from altered personality are far from having a similarly grave prognosis. 6. Duration: The persistence of any one idea, apart from its mode of development, for several months is a bad omen. 7. Hallucinations: Of these, as of delusions, it may be said that duration, slow growth, and fixity exercise great weight in prognosis; aural, olfactory, and those of common sensation are said to be the more unfavourable. Their persistence, when discordant with the general emotional tone, is also adverse.

**Varieties of Insanity.**—A few words may be said about some different forms of insanity as to their respective prognoses. (*a*) General paralysis may be at once dismissed from consideration, as practically having but one outcome. (*b*) Epileptic insanity: The prognosis in this form is rendered much less favourable by the epilepsy. To some extent it is dependent upon the successful treatment of the latter. (*c*) Senile: The prognosis is not necessarily grave unless advanced organic changes have occurred in the brain or elsewhere. Bad indications are afforded by melancholic attacks with serious lesions of memory, refusal of food, &c. Recovery is, however, rarely complete under any circumstances. (*d*) Adolescence: The prognosis in this is that before given of hereditary and relapsing cases. Long continued masturbation is a bad element. Recovery not ensuing by the ninth month in males and tenth in females may give cause for anxiety. (*e*) Climacteric: Cases occurring at over 50 years of age show a lessened recovery rate. Other adverse elements in prognosis are previous alcoholic habits, ideas of suspicion and persecution, eroticism arising during the attack. (*f*) Puerperal: The comparatively few cases which do not recover show some such circumstances as very slow onset, with physical exhaustion, severe puerperal fever, the development of delusions of persecution, exaltation, of persistent hallucinations, prolonged insomnia, alternations of excitement and depression, or physical improvement, and the return of menstruation before mental progress. Prognosis is also less hopeful when treatment has been delayed, or when the patient is over 30 years of age. After the attack there is liability to permanent instability of character, deterioration in morals, habits, and behaviour, and where hereditary taint is present there is much likelihood of a relapse at the next childbirth. (*g*) Post febrile insanity: The delirium of such fevers as typhoid, small-pox, and scarlatina, if severe, may pass into definite mental disorder if predisposition exist. Influenza may also precipitate a latent neurosis, and some degree of incurable dementia is prone to follow these forms. (*h*) Impulsive insanity: The prognosis must be unfavourable not as regards temporary recovery, but as to the impossibility of forecasting when a return of the outburst will occur, and what serious mischief may be the result. (*i*) Alcoholic insanity: The acute attacks of alcoholism following a more or less brief bout of drinking indicate hereditary instability, and, therefore, a likelihood of quick recovery, and probable relapse on repetition of the indulgence. In these cases especially unfavourable symptoms are afforded by sudden exhibitions of suicidal or aggressive tendencies, exaltation, of painful hallucinations, suspicions, convulsive attacks. Once chronic alcoholic insanity is established, with its ideas of persecution, hallucinations (cutaneous and aural), lapses of memory, and motor spasms, prognosis is most unfavourable, and, with the appearance of exaltation or change in personality, becomes hopeless. Senile-like reductions occurring rapidly in a person beyond middle-age after long years of chronic intemperance have a most gloomy significance. (*j*) Stupor: The varieties of stupor must be separated to form an opinion as to recovery. That following epilepsy and G.P. need not detain us. That succeeding acute disorder is less favourable than primary stupor, especially the melancholic form (more properly called melancholia attonita for the resistive and impulsive acts, often met with, indicate hidden delusions of a painful character not seldom terminating in attempts at suicide). About half these cases drift into chronic melancholia, weak-mindedness, or else imperfectly recover. Primary stupor is not unfavourable unless complicated by phthisis or other source of malnutrition, or unless heredity is strongly operative. The apparent degradation of the patient is no clue to prognosis. True dementia, which is secondary and gradually developed out of acute insanities, is of course not recoverable. Remembering the high recovery rate in hereditarily degenerate cases it will be evident that the dementia occurring in their earlier attacks is highly ominous, whilst again, that dementia, whether primary or secondary, attacking persons who have cardiac and arterial degeneration, phthisis, cancer, chronic Bright's disease, syphilis, alcoholism, coarse cerebral or meningeal lesions, &c., must threaten mental extinction.

